# Tobacco smoking by medical students: KAP study

**DOI:** 10.4103/1817-1737.69121

**Published:** 2010

**Authors:** Sourabh Aggarwal

**Affiliations:** *University College of Medical Sciences, New Delhi, India. E-mail: drsourabh79@gmail.com*

Sir,

I read the article “Knowledge, attitude and practice of tobacco smoking by medical students”[1] by Al-Haqwi AI *et al*. with interest. The authors would have made a commendable effort to study such a topic in medical students and their efforts should be appreciated. I would like to share my views to the same.

It is indeed a serious problem that up to 24% of the male students in medical colleges smoke tobacco. The gravity of the situation needs to be understood and serious measures should be taken by the concerned authorities. Because most of these habits start in the hostel, strict restrictions should be imposed on students in hostels to curb this menace besides offering counseling to students in this regard. It is of utmost importance as these students are physicians of the future and they have to impart health education in the future and serve as a role model for their patients in time to come, and such unhealthy habits can have a bad impact on the health of the society in a bigger prospective. It would have been interesting to notice the pattern of use of smokeless tobacco among medical students. Nonetheless, the authors have done a great job, which needs to be applauded.
